# Fe-Doping Effect on Thermoelectric Properties of *p*-Type Bi_0.48_Sb_1.52_Te_3_

**DOI:** 10.3390/ma8030959

**Published:** 2015-03-05

**Authors:** Hyeona Mun, Kyu Hyoung Lee, Suk Jun Kim, Jong-Young Kim, Jeong Hoon Lee, Jae-Hong Lim, Hee Jung Park, Jong Wook Roh, Sung Wng Kim

**Affiliations:** 1 Department of Energy Science, Sungkyunkwan University, Suwon 440-746, Korea; E-Mail: vzkzmmha@skku.edu; 2 Department of Nano Applied Engineering, Kangwon National University, Chuncheon 200-701, Korea; E-Mail: khlee2014@kangwon.ac.kr; 3 School of Energy, Materials and Chemical Engineering, Korea University of Technology and Education, Cheonan 330-708, Korea; E-Mail: skim@koreatech.ac.kr; 4 Icheon Branch, Korea Institute of Ceramic Engineering and Technology, Icheon 467-843, Korea; E-Mail: jykim@kicet.re.kr; 5 Department of Electrical Engineering, Kwangwoon University, Seoul 139-701, Korea; E-Mail: jhlee@kw.ac.kr; 6 Electrochemistry Department, Korea Institute of Materials Science, Changwon 641-010, Korea; E-Mail: lim@kims.re.kr; 7 Materials R&D Center, Samsung Advanced Institute of Technology, Samsung Electronics, Suwon 443-370, Korea; E-Mail: hj2007.park@samsung.com

**Keywords:** doping, Bi_2_Te_3_, thermoelectric, raw material, lattice thermal conductivity

## Abstract

The substitutional doping approach has been shown to be an effective strategy to improve *ZT* of Bi_2_Te_3_-based thermoelectric raw materials. We herein report the Fe-doping effects on electronic and thermal transport properties of polycrystalline bulks of *p*-type Bi_0.48_Sb_1.52_Te_3_. After a small amount of Fe-doping on Bi/Sb-sites, the power factor could be enhanced due to the optimization of carrier concentration. Additionally, lattice thermal conductivity was reduced by the intensified point-defect phonon scattering originating from the mass difference between the host atoms (Bi/Sb) and dopants (Fe). An enhanced *ZT* of 1.09 at 300 K was obtained in 1.0 at% Fe-doped Bi_0.48_Sb_1.52_Te_3_ by these synergetic effects.

## 1. Introduction

The thermoelectric (TE) effect is the direct conversion of temperature difference to electric energy and vice-versa. Thus, it can be used to measure temperature, to cool objects, to heat them, or to generate electricity. However, current applications of TE devices are limited to small-scale refrigeration, high density cooling, or precise temperature control systems, mainly due to the low conversion efficiency of TE materials, which is evaluated in terms of a dimensionless figure merit *ZT* (=*σS*^2^*T*/*κ*, where *σ* is the electrical conductivity; *S* is the Seebeck coefficient; and *κ* is the total thermal conductivity at a given absolute temperature *T*).

Bi_2_Te_3_-based alloys (*p*-type Bi_2–*x*_Sb*_x_*Te_3_ and *n*-type Bi_2_Te_3–*y*_Se*_y_*) are the best TE materials near to room temperature, and are widely used for cooling applications. The compositions of commercialized *p*-type and *n*-type ingots are near Bi_0.5_Sb_1.5_Te_3_ and Bi_2_Te_2.7_Se_0.3_, respectively, and their *ZT* values are about 1.0 at 300 K [1,2]. Their polycrystalline bulk forms, especially for *p*-type materials, have been also developed because of the enhanced *ZT* and mechanical properties. For example, Xie *et al.* reported that enhanced *ZT* of 1.1 at 300 K could be obtained in polycrystalline Bi_0.52_Sb_1.48_Te_3_ made from pulverized ingot by spark plasma sintering (SPS) [3]. However, this material cannot be used for TE cooling module due to its low *σ* value (~670 S·cm^−1^ at 300 K). For cooling application, *σ* value at 300 K should be controlled in value above 900 S·cm^−1^ to optimize the heat balance among Peltier heat, Joule heat, and heat conduction.

It is well experimentally established that the *σ* values of *p*-type Bi_2–*x*_Sb*_x_*Te_3_ can be controlled by adjusting the ratio of Bi to Sb; *σ* values at 300 K could be controlled from 670 S·cm^−1^ (Bi_0.52_Sb_1.48_Te_3_) [3] to 1040 S·cm^−1^ (Bi_0.4_Sb_1.6_Te_3_) [4] in polycrystalline bulks. By this compositional tuning, we could obtain simultaneously enhanced *ZT* (~1.07 at 360 K) and appropriate *σ* (~980 S·cm^−1^ at 300 K) in Bi_0.42_Sb_1.58_Te_3_. This material is a promising candidate for the application of TE power generation from low grade heat (<100 °C) because the maximum *ZT* temperature shifts from 300 K to 360 K. Another approach to control *σ* value of Bi_2–*x*_Sb*_x_*Te_3_ is substitutional doping on Bi/Sb-site, which causes variation in carrier concentration (*n*) and/or modification of the density of states (DOS) [4,5,6,7,8].

In the present study, Fe-doped Bi_0.48_Sb_1.52_Te_3_ (Fe-B_0.48_ST, Bi_0.48_Sb_1.52–*x*_Fe*_x_*Te_3_, *x* = 0, 0.01, 0.02, 0.04) polycrystalline bulks were prepared by melting and SPS technique in order to clarify the effect of Fe-doping on the transport properties of *p*-type Bi_0.48_Sb_1.52_Te_3_ (BST). We investigated their TE properties and demonstrated the origin of the enhancement of electronic and thermal transport properties.

## 2. Results and Discussion

**Figure 1:**
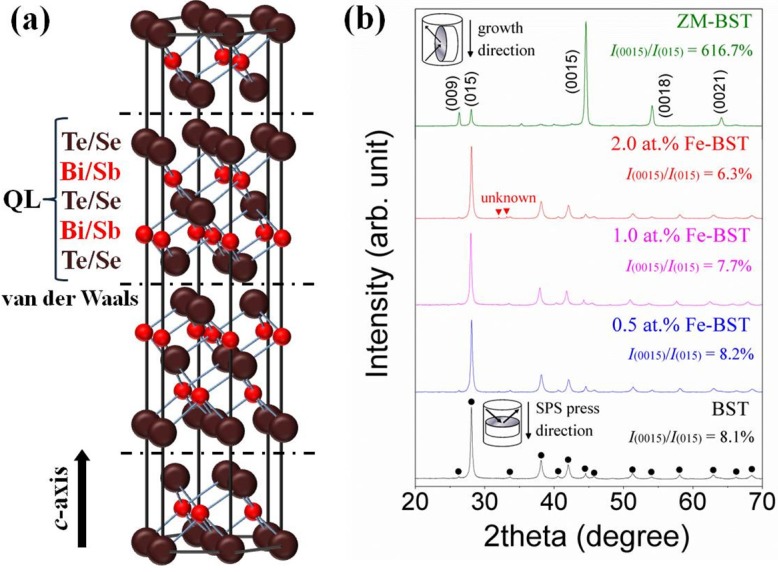
(**a**) Crystal structure of Bi_2_Te_3_-based thermoelectric materials; (**b**) X-ray diffraction (XRD) patterns of the zone-melted ingot of Bi_0.48_Sb_1.52_Te_3_ (ZM-BST) sample in the plane parallel to the growth direction and for BST and Fe-BST samples in the planes perpendicular to the spark plasma sintering (SPS) press direction.

Figure 1a represents the schematic structure of Bi_2_Te_3_-based TE alloys. Five atomic planes of Te(Se)-Bi(Sb)-Te(Se)-Bi(Sb)-Te(Se) compose quintuple layer (QL) along the *c*-axis. Because of the weak van der Waals bonding between QLs, Bi_2_Te_3_-based alloys can be preferentially grown into 2-dimensional structures by controlled crystal growth technique. Figure 1b shows the X-ray diffraction (XRD) patterns for ZM-BST sample in the plane parallel to the growth direction and for BST and Fe-BST samples in the planes perpendicular to the SPS press direction, respectively, as shown in the insets of Figure 1b.

**Figure 2:**
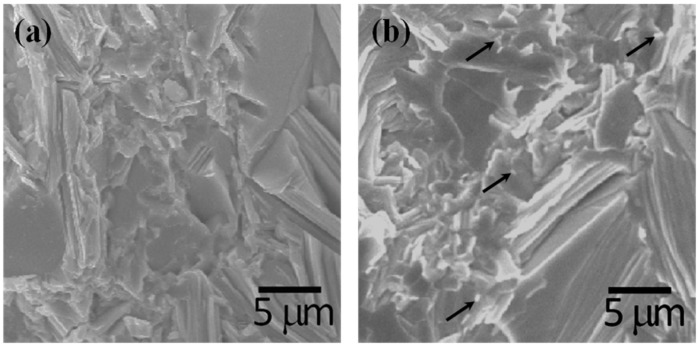
SEM images of fractured surface of (**a**) BST and (**b**) 2.0 at% Fe-BST samples.

For all samples, the structure is of the rhombohedral $R\bar{3}m.$ No other diffraction peaks corresponding to secondary or other structural phases are detected in ZM-BST, BST, and 0.5 at% and 1.0 at% Fe-BSTs, while a small amount of impurity peaks were found in 2.0 at% Fe-BST. As shown in Figure 1b, there is significant orientation in ZM-BST. The relative intensities of the {00*l*} planes including (009), (0015), (0018), and (0021) are much stronger than those of polycrystalline samples (BST and Fe-BSTs). We calculated the ratios *I*_(0015)_/*I*_(015)_ of the integrated intensity of (0015) to (015) and represented them in the inset of Figure 1b to evaluate the grain orientation anisotropy [9]. All samples show the anisotropy in crystal structure; however, the degree of anisotropic orientation is not significant in SPS consolidated polycrystalline samples. The *I*_(0015)_/*I*_(015)_ value for ZM-BST (616.7%) is much higher than those for BST and Fe-BSTs (6.3%–8.2%). Figure 2a,b shows the SEM images of the fractured surface of SPS consolidated BST and 2.0 at% Fe-BST samples, respectively. From the SEM images, we find that they are consistent with the XRD result: (1) a few plate-like grains with preferred orientation are clearly observed, which suggests the increased intensities of the {00*l*} planes and (2) a small amount of secondary phase is observed in 2.0 at% Fe-BST (arrows). The average grain size is about 5–10 μm even though there are many grains smaller than 5 μm.

**Figure 3:**
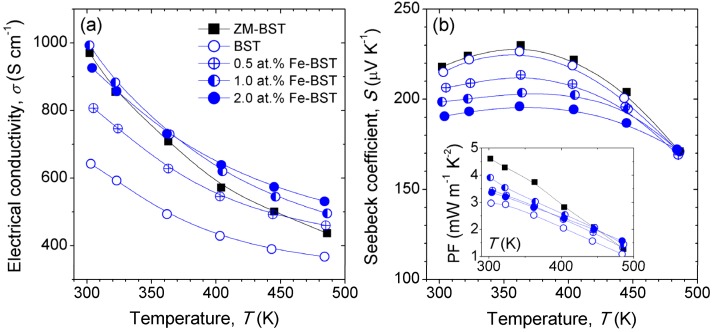
Temperature dependence of (**a**) the electrical conductivity (*σ*) and (**b**) the Seebeck coefficient (*S*) of ZM-BST, BST, and Fe-BTS samples. Inset shows the power factor (*σ*·*S*^2^) values.

In order to clarify the effects of Fe-doping on TE properties of BST, we measured the electronic transport properties. Figure 3 shows the temperature dependence of *σ* and *S* values for ZM-BST, reference BST, and Fe-BST samples. The value of *σ* at 300 K for ZM-BST is about 970 S·cm^−1^, while that for BST is 640 S·cm^−1^. Because the *n* value of ZM-BST (~2.36 × 10^19^ cm^−3^) is almost same as that of BST (~2.47 × 10^19^·cm^−3^), higher *σ* value of ZM-BST mainly due to the higher *μ* (~256 cm^2^·V^−1^·s^−1^) than that of BST (~161 cm^2^·V^−1^·s^−1^) originated from the significant anisotropy. On the other hand, *σ* values of BST increases with increasing amount of Fe-doping, suggesting that Fe acts as scattering center for electron conduction, while rather decreases in 2.0 at% Fe-BST owing to the decrease in *μ* (~130 cm^2^·V^−1^·s^−1^) in the presence of secondary phases. Comparable *σ* value of 993 S·cm^−1^ to that of ZM-BST (970 S·cm^−1^) can be obtained in polycrystalline Fe-BST by doping of 1.0 at% Fe. Figure 3b shows the temperature dependence of *S* for ZM-BST, reference BST, and Fe-BST samples. The *S* values of the Fe-BSTs decreases with increasing Fe-doping content because of the increase in *n* (3.12 × 10^19^ cm^−3^ for 0.5 at% Fe-BST, 3.78 × 10^19^ cm^−3^ for 1.0 at% Fe-BST, and 4.38 × 10^19^ cm^−3^ for 2.0 at% Fe-BST), while exhibit a moderate decrease with temperature especially above 360 K. The power factor (PF, *σ*·*S*^2^) values increase by Fe-doping with the tuning of *n*. For SPS consolidated samples, the largest PF value of 3.91 mW·m^−1^·K^−2^ at 300 K is obtained in 1.0 at% Fe-BST (inset of Figure 3b).

**Figure 4:**
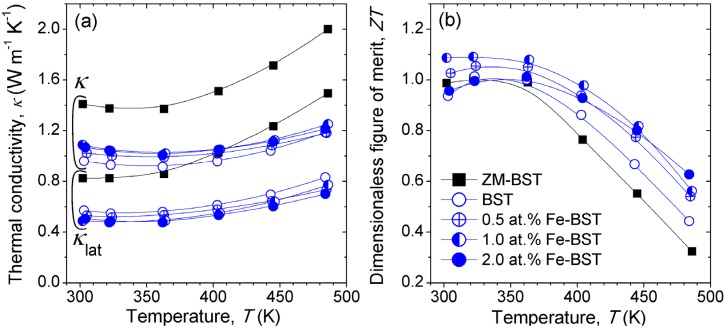
Temperature dependence of (**a**) the thermal conductivity (*κ*) and lattice thermal conductivity (*κ*_lat_) and (**b**) dimensionless of figure of merit *ZT* of ZM-BST, BST, and Fe-BTS samples.

Figure 4a shows the temperature dependence of the *κ* for ZM-BST, reference BST, and Fe-BST samples. We evaluated the *κ*_lat_ values, and represented them in Figure 4a. The *κ*_lat_ values were calculated by subtraction of *κ*_ele_ from the total *κ* (*κ*_lat_ = *κ* − *κ*_ele_, where *κ*_ele_ is the electronic contribution of thermal conduction), and the *κ*_ele_ values was estimated using the Wiedemann-Franz law, *κ*_ele_ = *L*_0_*T* *σ*, in which *L*_0_ denotes the Lorenz number, taken to be 2 × 10^−8^ W·Ω·K^−2^, a value reasonable for Bi_2_Te_3_-based TE materials. Room temperature values of *κ*_lat_, ranging from 0.49 W·m^−1^·K^−1^ to 0.53 W·m^−1^·K^−1^, are found for Fe-BST samples. In comparison with reference BST (*κ*_lat_ ≈ 0.57 W·m^−1^·K^−1^ at 300 K), *κ*_lat_ decreases over 7% in the whole measured temperature range (300 K–480 K) by substitutional doping of Fe on Bi/Sb-site. This suggests that point-defect phonon scattering by mass difference between host atoms (Bi/Sb) and dopants (Fe) [10].

Figure 4b presents the temperature dependence of *ZT* for ZM-BST, reference BST, and Fe-BST samples. The *ZT* value at 300 K of 1.0, which is comparable to commercial *p*-type ingot, is obtained in ZM-BST. For polycrystalline bulk samples, peak *ZT* value is 1.09, which shows a 16% increase for 1.0 at% Fe-BST at 300 K compared to the reference BST sample. This high *ZT* value is thought to be a result of synergetic effects, including PF enhancement by tuning of *n* and *κ*_lat_ reduction by intensified point-defect phonon scattering.

## 3. Experimental Section

High-purity elemental Bi (99.999%, 5N Plus), Sb (99.999%, 5N Plus), Te (99.999%, 5N Plus), and Fe (99.999%, Sigma Aldrich) as starting materials. The zone-melted ingot of Bi_0.48_Sb_1.52_Te_3_ (ZM-BST) was prepared using Bridgman method. According to the formula of BST and 4 wt% excess Te, the mixed elements were loaded into quartz tube of 14 mm in diameter and vacuum sealed under 10^−4^ torr. Quartz tube was placed in the vertical gradient furnace, then the temperature of the hot zone was increased to 1073 K. After this, the quartz tube was slowly pulled down at the rate of 0.2 mm·h^−1^. The ZM-BST of 30 mm in length was obtained. The source ingots for BST and Fe-BST powders were fabricated by the conventional melting and quenching techniques. Quartz tubes containing the stoichiometric mixed elements were vacuum sealed and the contents were melted in a box furnace for 10 h at 1073 K for BST and 1273 K for Fe-BST, respectively, then water quenched. The ingots were ground using ball mill and consolidated by SPS using graphite die (diameter = 11 mm) under dynamic vacuum and with the application of 50 MPa of uniaxial pressure at 703 K. The relative densities of the SPS consolidated samples (11 mm in diameter and 13 mm in thickness) were found to range from 6.62 to 6.74 g·cm^−3^ (>96% of the theoretical value). X-ray diffraction (D/MAX-2500/PC, Rigaku, Sendagaya, Japan) analysis was performed for bulk samples to clarify the phase formation behavior. Room temperature Hall effect measurements were carried out in a constant magnetic field (1 T) and electric current (50 mA) using Keithley 7065 system. The bulk samples were structurally evaluated by performing scanning electron microscopy (SEM) using a JSM-7600F (JEOL, Akishima, Japan). The *S* and *σ* values from 300 K to 480 K were measured using a ZEM-3 system (ULVAC-RIKO, Yokohama, Japan). The *κ* values (*κ* = *ρ*_s_*C*_p_*λ*) were calculated separated measurements of sample density (*ρ*_s_), heat capacity (*C*_p_), and thermal diffusivity (*λ*):*C*_p_ values obtained using a PPMS (Quantum Design physical properties measurement system) were almost constant ~0.187 J·g^−1^·K^−1^ and *λ* values were collected by a laser-flash method (TC-9000, ULVAC-RIKO, Yokohama, Japan). We measured all TE properties in the perpendicular to the SPS press direction for polycrystalline BST and Fe-BST samples and in the parallel to the growth direction for ZM-BST sample.

## 4. Conclusions

In the present study, we fabricated the polycrystalline bulks of *p*-type Fe-doped Bi_0.48_Sb_1.52_Te_3_ by conventional melting and spark plasma sintering technique, and investigated their thermoelectric transport properties. The electronic transport properties including electrical conductivity and Seebeck coefficient of Bi_0.48_Sb_1.52_Te_3_ could be optimized by controlled doping of Fe on Bi/Sb-sites. Simultaneously, reduced lattice thermal conductivity was observed by the intensified phonon-defect phonon scattering in the presence of substitutional Fe impurities. Thus, the enhanced *ZT* of 1.09 was obtained at 300 K in Bi_0.48_Sb_1.52_Te_3_-based materials.
